# A MR conditional bioptome for MR guided myocardial biopsy: initial Experiences

**DOI:** 10.1186/1532-429X-15-S1-P40

**Published:** 2013-01-30

**Authors:** Mirja Neizel, Yang C Böring, Heinz-Werner Henke, Sascha Dahl, Sebastian Gruenig, Florian Bönner, Burkhard Sievers, Malte Kelm

**Affiliations:** 1 Cardiology, Pneumology and Angiology, University Hospital Düsseldorf, Düsseldorf, Germany; 2 Innovative Tomography Products, Bochum, Germany

## Background

Endomyocardial biopsy specimens can be critical to the diagnosis and treatment of various cardiomyopathic disorders. Since endomyocardial biopsy is normally guided by fluoroscopy, it depends on chance whether or not the biopsied specimens are obtained from abnormal areas and/or exhibit histological changes. Cardiac Magnetic Resonance (CMR) Imaging has the advantage of being able to visualize lesions by performing T2 weighted imaging and delayed enhancement imaging. Moreover, the feasibility of MR guided cardiac interventions has been demonstrated. However, there are still only limited MR conditional devices to realize MR guided myocardial biopsy in humans.

Therefore, the aim of this study was to evaluate a MR conditional myocardial bioptome for MR guided myocardial biopsy.

## Methods

The MR conditional bioptome was developed by Innovative Tomography Products (Bochum, Germany). For passive visualization of the bioptome magnetic markers were used. Different sizes of the magnetic markers were tested ex-vivo for optimal passive visualization. Moreover, the handling of the biopsy forceps was tested. All *ex vivo* experiments were performed using an interventional 1.5 Tesla MRI system (Achieva, Philips, Best, Netherlands). The laboratory was equipped with interventional in-room monitors to guide the procedure. For real time imaging we used a SSFP-sequence (TE 2.5, TR 1.25, flip angle 45°, slice thickness 8mm, matrix 128 x 128 reconstructed to 256 x 256 using zero-filling). The experiments were carried out on an ex-vivo swine heart connected with a vessel phantom in a water filled basin. A sheath inserted in the tub was used for easy introduction of the guiding catheter.

## Results

The different sizes of markers imposed artifacts in the range of factor 6 to 16 in magnification. The size of the artifacts differed in sagittal and transversal axes. After detailed evaluation a marker size with good visibility and moderate artifact (4x) was selected and four markers were placed at the distal end of the bioptome pull wire in 40 mm distance. Additionally two markers were brought to the tip of the bioptome which had good visibility and moderate magnification (33 x 25 mm). The guiding sheath of the bioptome was marked with one ring at the distal end resulting in a "cross"-like artifact. There was a good visibility of the bioptome all the way through the aortic phantom and could also be depicted excellent in the myocardium tissue. No problems of localization of the bioptome tip occurred in this setting. The bioptome could be opened easily in the myocardium and worked fine grasping myocardial tissue.

## Conclusions

Initial experience with a MR conditional bioptome did show excellent results for visibility and navigation of the instrument in the setting of real-time imaging. This is one step further towards MR-guided myocardial biopsy in the near future.

**Figure 1:**
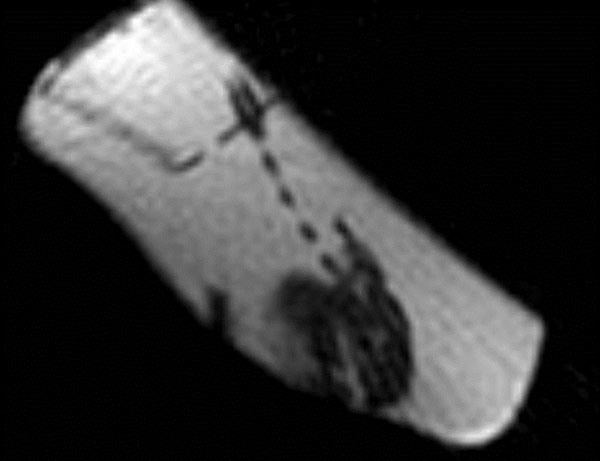


## Funding

No funding.

